# LncRNA H19 Regulates BMP2-Induced Hypertrophic Differentiation of Mesenchymal Stem Cells by Promoting Runx2 Phosphorylation

**DOI:** 10.3389/fcell.2020.00580

**Published:** 2020-07-29

**Authors:** Guangming Dai, Haozhuo Xiao, Chen Zhao, Hong Chen, Junyi Liao, Wei Huang

**Affiliations:** Department of Orthopaedic Surgery, The First Affiliated Hospital of Chongqing Medical University, Chongqing, China

**Keywords:** BMP2, lncRNA H19, MSCs, hypertrophic differentiation, cartilage tissue engineering

## Abstract

**Objectives:**

Bone morphogenetic protein 2 (BMP2) triggers hypertrophic differentiation after chondrogenic differentiation of mesenchymal stem cells (MSCs), which blocked the further application of BMP2-mediated cartilage tissue engineering. Here, we investigated the underlying mechanisms of BMP2-mediated hypertrophic differentiation of MSCs.

**Materials and Methods:**

*In vitro* and *in vivo* chondrogenic differentiation models of MSCs were constructed. The expression of H19 in mouse limb was detected by fluorescence *in situ* hybridization (FISH) analysis. Transgenes BMP2, H19 silencing, and overexpression were expressed by adenoviral vectors. Gene expression was determined by reverse transcription and quantitative real-time PCR (RT-qPCR), Western blot, and immunohistochemistry. Correlations between H19 expressions and other parameters were calculated with Spearman’s correlation coefficients. The combination of H19 and Runx2 was identified by RNA immunoprecipitation (RIP) analysis.

**Results:**

We identified that H19 expression level was highest in proliferative zone and decreased gradually from prehypertrophic zone to hypertrophic zone in mouse limbs. With the stimulation of BMP2, the highest expression level of H19 was followed after the peak expression level of Sox9; meanwhile, H19 expression levels were positively correlated with chondrogenic differentiation markers, especially in the late stage of BMP2 stimulation, and negatively correlated with hypertrophic differentiation markers. Our further experiments found that silencing H19 promoted BMP2-triggered hypertrophic differentiation through *in vitro* and *in vivo* tests, which indicated the essential role of H19 for maintaining the phenotype of BMP2-induced chondrocytes. In mechanism, we characterized that H19 regulated BMP2-mediated hypertrophic differentiation of MSCs by promoting the phosphorylation of Runx2.

**Conclusion:**

These findings suggested that H19 regulates BMP2-induced hypertrophic differentiation of MSCs by promoting the phosphorylation of Runx2.

## Introduction

Stem-cell-based and gene-enhanced tissue engineered cartilage is promising in the treatment of cartilaginous pathologies, especially traumatic cartilage defects (Bishop et al., 2017; Canadas et al., 2018; Wang et al., 2019). Mesenchymal stem cells (MSCs) hold the potential for osteogenic, chondrogenic, adipogenic differentiation, etc., owing to the fact that MSCs are easy to isolate, stable in expressing exogenous genes, abundant in source, and were identified as ideal seed cells for regenerative medicine (Canadas et al., 2018; Kim et al., 2019; Mamidi et al., 2016; Wang et al., 2019). Therefore, it is essential to guide MSC chondrogenic differentiation for the construction of tissue engineering cartilage.

Bone morphogenetic protein 2 (BMP2), a member of the transforming growth factor beta (TGF-β) superfamily, is characterized as one of the most effective growth factors to induce MSC chondrogenic differentiation (Kovermann et al., 2019; Miyazono et al., 2010; Munsell et al., 2018; Pan et al., 2008; Zhou et al., 2016). However, BMP2 is also known to induce MSC osteogenic differentiation and stimulate hypertrophic differentiation after chondrogenic differentiation, which go against maintaining of BMP2-induced cartilage phenotype (An et al., 2010; Liao et al., 2014; Zhou et al., 2016). Our previous studies found that BMP2 induced MSC chondrogenic differentiation by upregulating the expression of Sox9; as the key transcription factor of chondrogenesis, overexpression of Sox9 potentiated BMP2-mediated chondrogenic differentiation and inhibited BMP2-induced osteogenic differentiation (Liao et al., 2014; Zhou et al., 2016). However, BMP2 triggered hypertrophic differentiation process after chondrogenic differentiation still blocked the further application of BMP2-mediated cartilage engineering (Liao et al., 2014; Nasrabadi et al., 2018; Zhou et al., 2016). Hence, it is important to clarify the mechanisms underlying BMP2-mediated hypertrophic differentiation of MSCs.

With the development of next-generation sequencing technologies, it is confirmed that over 80% of human genome is transcribed; however, only ∼2% of human genome is transcribed into messenger RNA (mRNA), which indicates the pervasiveness of non-coding RNAs (ncRNAs) (Clark et al., 2011; Djebali et al., 2012; Pettersson et al., 2009). Recently, ncRNAs especially long non-coding RNAs (lncRNAs), which are identified as non-protein coding transcripts longer than 200 nucleotides, are characterized as regulatory RNAs and are involved in many physiological and/or pathological processes (Morris and Mattick, 2014; Quinn and Chang, 2016; Sanbonmatsu, 2016). As regulatory RNAs, lncRNAs were reported to serve as competition endogenous RNA (ceRNA), primary microRNA precursor, modular scaffold of histone modification, mRNA decay controller, functional protein regulator, etc. (Cai and Cullen, 2007; Kim and Shiekhattar, 2016; Morris and Mattick, 2014; Qi et al., 2019; Tsai et al., 2010). LncRNA H19 (H19), which was first isolated and reported in 1980s by four different laboratories, was identified as one of the first imprinted genes and lncRNAs (Cai and Cullen, 2007; Gabory et al., 2006; Hao et al., 1993; Liu et al., 2017; Moulton et al., 1994a). In the past several decades, H19 was known to regulate diverse cellular processes, including tumorigenesis, embryo growth, stem cell differentiation, etc. (Gabory et al., 2010; Hao et al., 1993; Liu et al., 2017; Moulton et al., 1994b). Meanwhile, evidence have shown that H19 was involved in MSC chondrogenic differentiation (Dudek et al., 2010; Liu et al., 2017; Pang et al., 2019). Dudek et al. (2010) reported that H19 and H19-encoded miR675 are essential for the production of Col2α. Pang et al. characterized that H19 is indispensable for the cartilage differentiation of stem cells. On the basis of our previous studies (Liao et al., 2017b), we speculated the regulatory function of H19 in BMP2-mediated chondrogenic differentiation of MSCs.

In the present study, we investigated the function of H19 in BMP2-mediated chondrogenic and hypertrophic differentiation of MSCs. We found a peak expression level of H19 after the crest stage of Sox9, and the expression levels of H19 were positively correlated with BMP2-mediated expression levels of chondrogenic differentiation markers, especially in the late stage. Our further experiments found that silencing H19 promoted BMP2-triggered hypertrophic differentiation, which indicated the essential role of H19 for maintaining the phenotype of BMP2-induced chondrocytes. In mechanism, we characterized that H19 can directly bind with Runx2 protein and promote Runx2 phosphorylation, which inhibited the function of Runx2. These findings applied a new version for the understanding of BMP2-mediated hypertrophic differentiation, which is beneficial for the construction of BMP2-mediated cartilage tissue engineering.

## Materials and Methods

### Ethics Statement

All animal protocols were approved by the Ethical Committee of The First Affiliated Hospital of Chongqing Medical University. All surgical operations were done under proper anesthesia; animals were kept in independent cages with standard conditions until it is confirmed that they recovered from anesthesia without pain. At the indicated time points, mice were euthanized by overdose intraperitoneal pentobarbital sodium (Sigma-Aldrich, United States) injection. All efforts were made to minimize the suffering of the animals; the ectopic masses were retrieved from the injection sites of the node mice after confirming that the mice were not breathing, have no heartbeat and with dilated pupils.

### Cell Culture and Chemicals

The human embryonic kidney (HEK) 293 and mouse bone marrow MSC C3H10T1/2 cell lines were obtained from the American Type Culture Collection (ATCC, Manassas, VA, United States). Cell lines were preserved in complete Dulbecco’s modified Eagle’s medium (DMEM, Hyclone, China), supplemented with 10% fetal bovine serum (FBS, Gibco, Australia), 100 U/ml penicillin, and 100 mg/ml streptomycin, maintained at 37°C in a humidified 5% carbon dioxide (CO_2_) atmosphere. Unless indicated otherwise, all chemicals were purchased from Sigma-Aldrich or Corning.

### Fluorescence *in situ* Hybridization

Fetal mouse limbs at embryonic 14.5 day were harvested, fixed overnight in diethyl pyrocarbonate (DEPC)-treated 4% paraformaldehyde (Servicebio, Wuhan, China), and embedded in paraffin. Then, serial 5-μm-thick sections were obtained and deparaffinized in xylene and rehydrated in graded ethanol and RNase-free deionized Millipore water (Invitrogen, CA, United States). The hybridization was performed as describe previously (Li et al., 2018). Prior to hybridization, tissue sections were pretreated with boiled target retrieval buffer supplied in the RNAscope kit (Invitrogen, CA, United States) for 15 min. Then, sections were hybridized for 3 h at 50°C in a hybridization oven with a mixture containing the hybridization buffer supplied in the kit and the probes for mouse H19 that were synthesized by Ribobio (Guangzhou, China) tagged with Cy3, followed by successive incubations and washing accordingly. Finally, slides were mounted with the antifade mounting media containing 4′,6-diamidino-2-phenylindole (DAPI, Vector Lab, Inc., Burlingame, CA, United States). The microscopy images of the sections were acquired with a fluorescence microscope (Olympus, United States). Cy3 (H19) and DAPI (nuclei) were excited at 561 and 405 nm, respectively. As for the analysis of fluorescence intensity, three high-power field of view were randomly selected in each area, and the optical density value per cells in each field was calculated by ImageJ Pro and calibrated relative to the background of the field. Then, the average cell optical density (by dividing nuclei numbers) of each area was calculated, and figure was drawn by GraphPad Prism software. The sequence of H19 probe is 5′-Cy3/*cagttgccctcagacggagatggacg*/Cy3-3′. H19 positive control for FISH analysis was shown in Supplementary Figure 1A.

### Construction and Generation of Recombinant Adenoviral Vectors AdBMP2, AdGFP, AdH19, and AdsimH19

Recombinant adenoviruses were generated using AdEasy technology as described previously (Deng et al., 2014; He et al., 1998; Lee et al., 2017; Luo et al., 2007); AdBMP2 was previously characterized (Liao et al., 2014; Zhou et al., 2015; Zhou et al., 2016), and AdGFP was used as a mock virus control. Briefly, the coding region of human BMP2, and the full-length transcript of mouse H19, were PCR amplified and subcloned into an adenoviral shuttle vector and used to generate recombinant adenoviral vectors; recombinant adenoviral vectors containing BMP2 or H19 were subsequently used to generate recombinant adenoviruses in HEK-293 cells. For making AdsimH19, three small interfering RNAs (siRNAs) targeting mouse H19 were simultaneously assembled to an adenoviral shuttle vector using the Gibson Assembly system as described (Deng et al., 2014; Liao et al., 2017b). AdBMP2 also expresses green fluorescent protein (GFP), whereas AdsimH19 expresses red fluorescent protein (RFP) as a marker for monitoring infection efficiency.

### Chondrogenic Differentiation of MSCs in Micromass Culture

To induce chondrogenic differentiation, micromass culture was used to mimic the condensation of MSCs as previously described (Liao et al., 2014). C3H10T1/2 cells were seeded at 60% confluence and infected with AdGFP, AdBMP2, or/and AdsimH19. Twenty-four hours after infection, cells were harvested and resuspended in high density (∼10^5^ per 25 μl medium), which were subsequently added at the center of each well in the 12-well plates and then incubated in CO_2_ incubator. One hour after incubation, 2–3 ml complete DMEM was added to each well; half medium was replaced every 3 days.

### RNA Isolation and RT-qPCR, RT Semiquantitative PCR

Total RNA was isolated with TRIZOL reagent (Invitrogen, CA, United States) according to the manufacturer’s instructions and subjected to reverse transcription reactions using PrimeScript RT reagent kit (Takara, Dalian, China). The quantitative PCR analysis was carried out using the CFX96 Real-Time PCR Detection System (Bio-Rad, CA, United States) with SYBR premix Ex Taq II kit (Takara, Dalian, China) according to the manufacturer’s instructions. Programs for real-time PCR are as follows: 95°C for 30 s, 95°C for 5 s, and 60°C for 30 s, repeating 40 cycles. Gapdh was used as a reference gene. The melting curves did not detect any non-specific amplification. All sample values were normalized to Gapdh expression by using the 2^–△^^△^^*Ct*^ method; primer efficiency correction was done and used to corrected amplification efficiency, Gradient concentration DNA samples were used for the normalization. The PCR primer sequences are listed in Table 1.

**TABLE 1:** Primer oligonucleotide sequences used for PCR.

Gene	Forward primer (5′–3′)	Reverse primer (5′–3′)
BMP2	ACCAGACTATTGGACACCAG	AATCCTCACATGTCTCTTGG
H19 for RT-qPCR	CAGAGTCCGTGGCCAAGG	CGCCTTCAGTGACTGGCA
H19 for RT-PCR	TATGCCCTAACCGCTCAGTC	AGACACCGATCACTGCTCC
SOX9	AGCTCAACCAGACCCTGAGAA	TCCCAGCAATCGTTACCTTC
Collagen 2α1	CAACACAATCCATTGCGAAC	TCTGCCCAGTTCAGGTCTCT
Aggrecan	TGGCTTCTGGAGACAGGACT	TTCTGCTGTCTGGGTCTCCT
MMP13	CTTTGGCTTAGAGGTGACTGG	AGGCACTCCACATCTTGGTTT
Collagen 10α1	CATGCCTGATGGCTTCATAAA	AAGCAGACACGGGCATACCT
MMP9	TTGACAGCGACAAGAAGTGG	CCCTCAGTGAAGCGGTACAT
Adamts5	CCTGCCCACCCAATGGTAAA	CCACATAGTAGCCTGTGCCC
GAPDH	CTACACTGAGGACCAGGTTGTCT	TTGTCATACCAGGAAATGAGCTT
β-actin	AGCCTCGCCTTTGCCGA	CTGGTGCCTGGGGCG

The semiquantitative PCR was preformed using premix Taq^TM^ (Takara, Dalian, China) kit, with the following programs: 92°C for 3 min for one cycle; 92°C for 30 s, 55°C for 30 s, and 72°C for 30 s, for 35 cycles. Then, PCR products were electrophoresed on 1% agarose gels (Invitrogen, CA, United States) and visualized by UV light.

### Western Blot Analysis

Protein extraction was performed by using 2% sodium dodecyl sulfate (SDS) lysis buffer that including 100 mM Tris–HCl, 100 mM β-mercaptoethanol, and protease and phosphatase inhibitors (Roche, United States). Total protein was denatured via boiling and determined using a BCA protein assay kit (Beyotime, Beijing, China). Equivalent amounts of protein were electrophoresed on 5–10% Bis-Tris gels (Life Technologies, MA, United States) and transferred to polyvinylidene fluoride membranes (PVDF, Millipore, MA, United States). Membranes were blocked with 5% skimmed milk for 1 h at room temperature and incubated with primary antibodies to collagen 10α1, MMP13, Runx2, phosphor-Runx2, and β-actin overnight (rabbit anti-collagen 10α1 and MMP13, 1:1,000, Abcam, Cambridge, United States; rabbit anti-Runx2 and β-actin, 1:1,000, Cell Signaling Technology, MA, United States; rabbit antiphosphor-Runx2, 1:1,500, Affinity Biosciences, United States). Following this, the membranes were incubated with corresponding secondary antibody conjugated with horseradish peroxidase (HRP, goat antirabbit secondary antibody, 1:1,000, Cell Signaling Technology, MA, United States). The blots were displayed with Immobilon Western Chemiluminescent HRP Substrate (Millipore, MA, United States). Relative protein expression was analyzed by Image Lab software using β-actin as control.

### Subcutaneous Stem Cell Implantation

The use and care of animals in this study were approved by the Institutional Animal Care and Use Committee. All experimental procedures were carried out in accordance with the approved guidelines. Subcutaneous stem cell implantation procedure was performed as described (Liao et al., 2014; Liao et al., 2017a; Liao et al., 2017b). Briefly, the C3H10T1/2 cells were infected with AdGFP, AdBMP2, and/or AdsimBMP2. Twenty-four hours after infection, cells were collected and resuspended in DMEM at a density of ∼2 × 10^5^/μl (100 μl each injection). The cells were injected subcutaneously into the flanks of athymic nude mice (*n* = 3/group, female, 5–6 weeks old).

At the indicated time points, animals were euthanized, and the ectopic masses were retrieved from injection sites and subjected to X-ray imaging system with automatic exposure under 45 kV, 500 mA. Then, the masses were fixed in 4% paraformaldehyde (Beyotime, Beijing, China) for 24 h at room temperature, decalcified in 0.5 M ethylenediaminetetraacetic acid (EDTA) at 4°C for 14 days and embedded in paraffin. Serial 5-μm-thick sections were obtained and followed by histological and other specialty staining evaluations.

### Histological Evaluation: Hematoxylin and Eosin, and Alcian Blue Staining

Sections were deparaffinized with xylene and rehydrated using graded ethanol. H&E and Alcian blue staining were performed using standard protocol as previously described (Liao et al., 2014; Zhou et al., 2015; Zhou et al., 2016). Briefly, the deparaffinized samples were first subjected to antigen retrieval and fixation. Then, sections were stained with hematoxylin and eosin (H&E) and Alcian blue staining. Histological evaluation was performed with the use of a light microscope (Olympus, Japan).

### Immunohistochemistry Assay

Generally, sections were deparaffinized with xylene, rehydrated using graded ethanol, treated with 3% H_2_O_2_ for 10 min to inhibit endogenous peroxidase activity, boiled in citrate buffer (pH 6.0) for 20 min at 95–100°C, and blocked with normal goat serum. Then, sections were incubated with primary antibody to MMP-13 (Santa Cruz Biotechnology Inc., Texas, United States, 1:300 dilution), Collagen 10α1 (Abcam, Cambridge, United States, 1:200 dilution), Runx2 (Cell signaling Technology, MA, United States, 1:200 dilution), and collagen 1α1 (Abcam, Cambridge, United States, 1:200 dilution) at 4°C overnight. After being washed, the sections were incubated with biotin-labeled secondary antibody for 30 min, followed by incubation with streptavidin–HRP conjugate for 20 min at room temperature. Staining without primary antibody was utilized as negative control. Staining without primary antibody was utilized as negative control (Supplementary Figure 1B). All photos were obtained by using a microscope (Olympus, Japan).

### RNA Immunoprecipitation Analysis

The RNA immunoprecipitation (RIP) analysis was done as described previously (Kallen et al., 2013; Yuan et al., 2014). C3H10 T1/2 cells were infected with AdH19 or AdGFP. Three days after infection, cells were lysed and subjected to RIP analysis with the use of Magna RIPTM RNA-Binding Protein Immunoprecipitation Kit (Millipore, Bedford, MA, United States) according to the manufacturer’s instructions. Briefly, prior to immunoprecipitation, magnetic beads were pretreated with RIP wash buffer supplied in the kit and incubated with Runx2 antibody (Cell Signaling Technology, MA, United States) for 30 min. Then, the magnetic bead-Runx2 composites were incubated with cell lysis supernatant and RIP immunoprecipitation buffer overnight at 4°C followed by successive washing with RIP wash buffer. After treatment with immunoprecipitation, the composites were detached by using proteinase K buffer. After that, the RNA fraction was isolated and subjected to perform qPCR analysis as described above.

### Statistical Analysis

All images were obtained by using a microscope (Olympus, Japan) and analyzed by ImageJ Pro. Data were expressed as mean ± standard deviation (SD) and analyzed with SPSS software (Version 21, IBM, United States). A one-way analysis of variance was performed to analyze inter- and intragroup differences when more than two groups were compared. A *t* test was used to compare between any two groups. The correlations between H19 expression and other parameters were calculated with Spearman’s correlation coefficients. A value of *P* < 0.05 was considered statistically significant.

## Results

### H19 Expression in Fetal Mouse Limb

**FIGURE 1:**
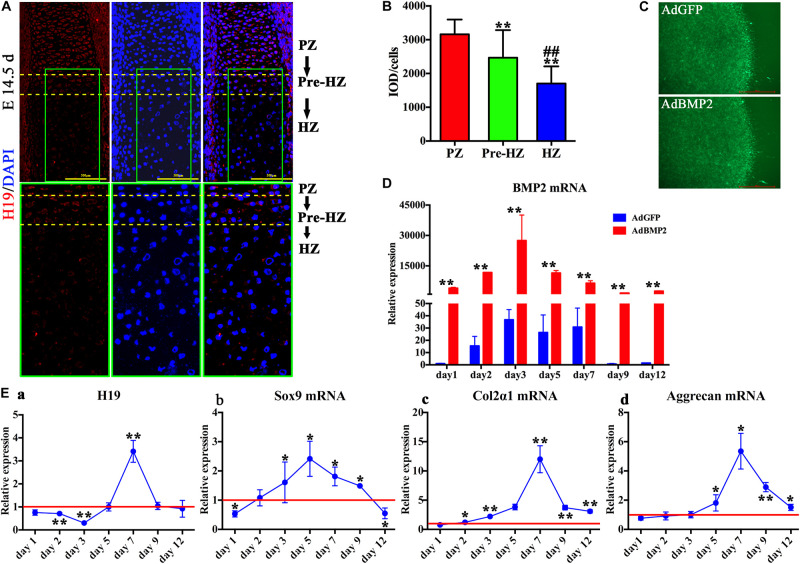
H19 expression in developing endochondral bones and chondrogenic differentiation of mesenchymal stem cells (MSCs) induced by bone morphogenetic protein 2 (BMP2). **(A)** H19 expression in developing endochondral bones. Detection of H19 by fluorescence *in situ* hybridization to sections of the developing radius at E14.5 day. Expression of H19 (Red) is mainly observed in the proliferative zone (PZ), decreased in the prehypertrophic zone (Pre-HZ), and in lowest level in the hypertrophic zone (HZ). Higher magnification images were listed in the lower panel; scale bar = 500 μm. **(B)** Quantitative analysis of positive H19 expression. **(C)** BMP2-induced chondrogenic differentiation of MSCs in micromass culture. C3H10T1/2 cells were infected with AdBMP2 or AdGFP and subjected to micromass culture. Green fluorescent protein (GFP) fluorescence fields were recorded at 3 days after infection, scale bar = 1,000 μm. **(D)** Adenovirus-mediated overexpression of BMP2. The expression level of BMP2 messenger RNA (mRNA) was detected by quantitative PCR (qPCR) from days 1 to 12 after the infection of AdBMP2; AdGFP was used as control. **(E)** BMP2-induced H19 and chondrogenic differentiation markers expression. Relative expression levels of H19 **(a)**, Sox9 mRNA **(b)**, collagen 2α1 mRNA **(c)**, and Aggrecan mRNA **(d)** were detected by reverse transcription and quantitative real-time PCR (RT-qPCR) at a series time (days 1, 2, 3, 5, 7, 9, and 12) with the stimulation of AdBMP2. AdGFP was used as control. The values of AdBMP2/AdGFP were shown (each assay was done in triplicate and/or carried out in three independent experiments at least). “**” and “##” mean *p* < 0.01 compared with PZ and Pre-HZ group, respectively in panel **(B)**; “*” and “**” respectively mean *p* < 0.05 and *p* < 0.01 compared with AdGFP group in panel **(D)** and **(E)**. DAPI, 4′, 6-diamidino-2-phenylindole, PZ, proliferative zone, Pre-HZ, pre-hypertrophic zone, HZ, hypertrophic zone.

To understand the function of H19 during the process of hypertrophic differentiation, we determined the expression of H19 in day 14.5 fetal mouse limb with the use of fluorescence *in situ* hybridization (FISH) technology. As shown in Figure 1A, the expression of H19 was highest in the proliferative zone, decreased in the prehypertrophic zone, and in lowest level in the hypertrophic zone in day 14.5 fetal mouse limb (Figure 1A, upper panel). Higher magnification (Figure 1A, lower panel) and H19-positive cells quantitative analysis (Figure 1B) showed the same trend. These results indicate that H19 may play a role in maintaining the phenotype of chondrocytes.

### BMP2-Induced H19 Expression and Chondrogenic Differentiation of MSCs in Micromass Culture

To mimic the process of MSCs condensation, C3H10T1/2 cells infected with AdBMP2 or AdGFP were subjected to micromass culture (Figure 1C). We first determined mRNA expression level of BMP2, as shown in Figure 1D, compared with AdGFP group, AdBMP2 dramatically increased the expression of BMP2 from day 1 to 12, which indicated that adenovirus-mediated overexpression of BMP2 was effective and sustained more than 12 days in micromass culture.

Second, we determined BMP2-induced expression of H19. As shown in Figure 1Ea, we found that the expression of H19 was downregulated by BMP2 from day 1 to 3, back to the basal level at day 5, then dramatically upregulated at day 7, and finally back to the basal level at days 9 and 12. These results indicated that H19 would function at the medial or late stage of BMP2-mediated MSC chondrogenic differentiation.

In addition, we detected the chondrogenic differentiation marker expression with the stimulation of BMP2. To be consistent with our previous work, we found that the expression level of Sox9 was upregulated by BMP2 from day 2 to 9 and showed a highest level at day 5 (Figure 1Eb). Meanwhile, Col2α and Aggrecan expressions decreased gradually from day 1 to 7 and showed a peak level at day 7, then back to the basal level gradually from day 9 to 12 (Figures 1Ec,d). Meanwhile, the data demonstrated that the peak expression level of H19 was followed after the crest expression level of Sox9 (Figure 1E).

Putting these data together, we infer that H19 may be acting as a regulatory RNA at medial or late stage of BMP2-induced chondrogenic differentiation of MSCs in micromass culture.

### BMP2-Induced H19 Expression Is Positively Correlated With Terminal Chondrogenic Differentiation Markers and Negatively Correlated With Hypertrophic Differentiation Markers

**FIGURE 2:**
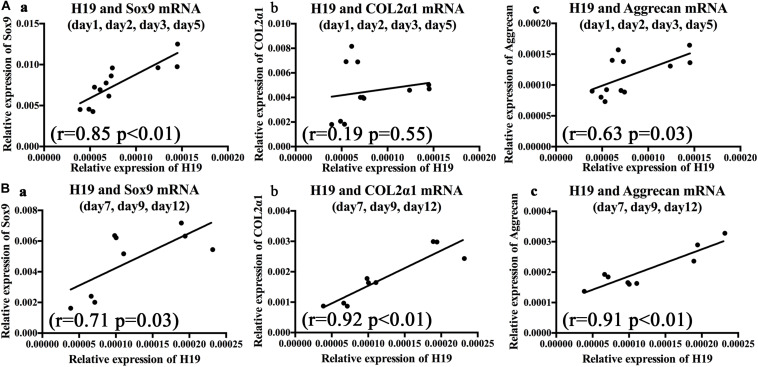
Bone morphogenetic protein 2 (BMP2)-induced H19 expression is positively correlated with terminal chondrogenic differentiation markers. The correlations between BMP2-induced H19 expression and Sox9 messenger RNA (mRNA), collagen 2α1 mRNA, and Aggrecan mRNA were analyzed from day 1 to 5 and day 7 to 12, respectively, with Spearman’s correlation coefficients, *r* means correlation coefficients. **(A)** During the early stage of BMP2’s stimulation (days 1–5) Sox9 (*r* = 0.85, *p* < 0.01) **(a)** and Aggrecan (*r* = 0.63, *p* = 0.03) **(c)** expression levels were positively corelated with H19 expression level; however, Col2α (*r* = 0.19, *p* = 0.55) **(b)** expression levels were not significantly correlated with H19 expression levels. **(B)** In the late stage of BMP2’s stimulation (days 7–12) Sox9 mRNA (*r* = 0.71, *p* = 0.03) **(a)**, Col2α mRNA (*r* = 0.92, *p* < 0.01) **(b)**, and Aggrecan mRNA (*r* = 0.91, *p* < 0.01) **(c)** expression levels were positively corelated with H19 expression levels. Each assay was done in triplicate and/or carried out in three independent experiments at least; mean value of each independent experiment was shown.

To further clarify the relationships between BMP2-induced H19 expression levels and chondrogenic or hypertrophic differentiation markers, correlation analysis was used to analyze the correlation between H19 expression levels and chondrogenic and hypertrophic differentiation markers with the stimulation of BMP2. As for the chondrogenic differentiation markers, on the basis of H19 expression level, we analyzed days 1–5 and days 7–12, respectively. As shown in Figure 2A, from days 1 to 5, there was no obvious correlation between H19 expression level and Col2α1 (*r* = 0.19, *P* = 0.55); however, H19 expression levels were positively correlated with key chondrogenic differentiation transcription factor Sox9 expression levels (*r* = 0.85, *P* < 0.01) and chondrogenic marker Aggrecan (*r* = 0.63, *p* = 0.03). What is interesting is that from day 7 to 12 (Figure 2B), H19 expression levels were positively correlated with the expression levels of Sox9 (*r* = 0.71, *P* = 0.03), Col2α1 (*r* = 0.92, *P* < 0.01), and Aggrecan (*r* = 0.91, *P* < 0.01). These data highly indicated that H19 might regulate BMP2-induced terminal chondrogenic differentiation.

**FIGURE 3:**
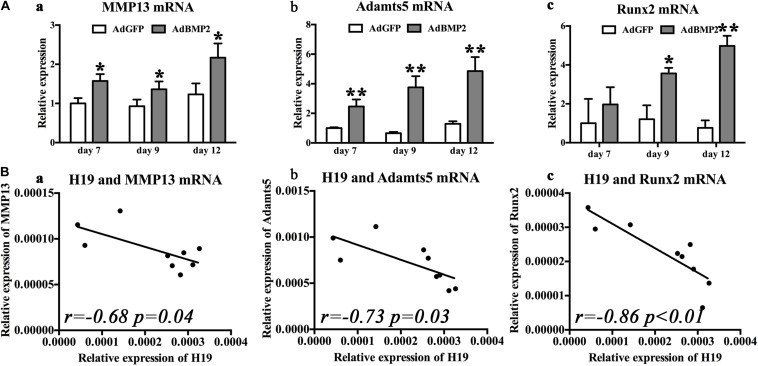
Bone morphogenetic protein 2 (BMP2) induced H19 expression is negatively correlated with hypertrophic differentiation markers. **(A)** BMP2-induced hypertrophic differentiation of mesenchymal stem cells (MSCs). Quantitative PCR (qPCR) analysis of MMP13 **(a)**, Adamts5 **(b)**, and Runx2 **(c)** messenger RNA (mRNA) expression levels at days 7, 9, and 12 with the stimulation of AdBMP2; AdGFP was used as control. **(B)** The correlations between BMP2-induced H19 expression level and hypertrophic differentiation markers expression levels. The correlations between H19 expression levels of MMP13 (*r* = −0.68, *p* = 0.04) **(a)**, Adamts5 (*r* = 0.73, *p* = 0.03) **(b)**, and Runx2 (*r* = −0.86, *p* < 0.01) **(c)** mRNA expression levels at days 7, 9, and 12 were calculated with Spearman’s correlation coefficients. “*” and “**” respectively mean *p* < 0.05 and *p* < 0.01 compared with AdGFP group; *r* means correlation coefficients. Each assay was done in triplicate and/or carried out in three independent experiments at least.

As for the hypertrophic differentiation markers, we first confirmed that, with the stimulation of BMP2, hypertrophic differentiation markers (MMP13, Adamts5, and Runx2) were significantly upregulated compared with control groups from day 7 to 12 (Figures 3Aa,Ba,Ca). Second, correlation analysis exhibited that H19 expression levels were negatively correlated with the expression levels of hypertrophic differentiation markers (MMP13, *r* = −0.68, *P* = 0.04; Adamts5, *r* = −0.73, *P* = 0.03) (Figure 3B) and key hypertrophic differentiation transcription factor Runx2 (*r* = −0.86, *P* < 0.01). Taken these data together, we deduced that H19 could play an important role in regulating BMP2-induced hypertrophic differentiation.

### Silencing of H19 Promoted BMP2-Induced Hypertrophic Differentiation of MSCs *in vitro* and *in vivo*

**FIGURE 4:**
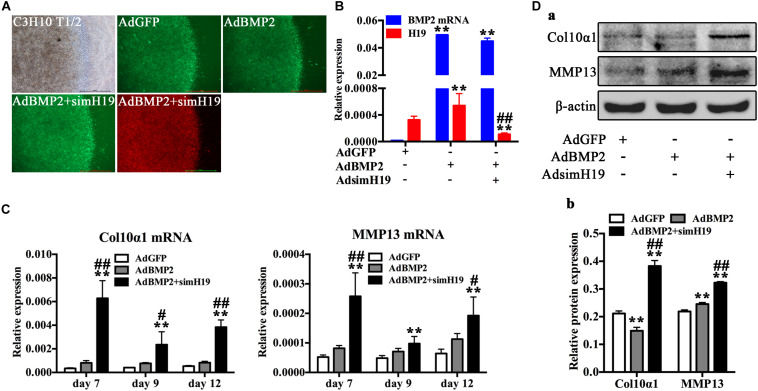
Silencing of H19 promoted bone morphogenetic protein 2 (BMP2)-induced hypertrophic differentiation of mesenchymal stem cells (MSCs) *in vitro*. **(A)** C3H10T1/2 cells infected with AdGFP, AdBMP2, AdBMP2, and AdsimH19 were subjected to micromass culture. Bright field, GFP (AdGFP, AdBMP2) and RFP (AdsimH19) fluorescence fields were recorded at day 3 after infection; the fluorescence indicated high efficiency in single or combination infection, scale bar = 1,000 μm. **(B)** Effective knockdown of mouse H19 expression. The expression level of H19 and BMP2 messenger RNA (mRNA) at day 3 in different groups were detected by reverse transcription and quantitative real-time PCR (RT-qPCR); AdsimH19 silences the expression of H19 without influencing the expression of BMP2. All samples were normalized with the reference gene Gapdh. Each assay condition was done in triplicate. **(C)** Silencing H19 promoted BMP2-induced collagen 10α1 and MMP13 mRNA expression. Subconfluent MSCs were infected with AdBMP9 or AdGFP and/or AdsimH19 and subjected to micromass culture. At the indicated time points, total RNA was isolated and subjected to qPCR analysis using primers for mouse collagen 10α1 and MMP13; each assay condition was done in triplicate. **(D)** Silencing H19 promoted BMP2-induced collagen 10α1 and MMP13 protein expression. Western blot for the expression of collagen 10α1 and MMP13 were conducted at day 7 after transduction of indicated recombinant adenoviruses **(a)**. Relative protein expression was analyzed by Image Lab software using β-actin as control **(b)**. “*” and “**” respectively mean *p* < 0.05 and *p* < 0.01 compared with AdGFP group; “#” and “##” respectively mean *p* < 0.05 and *p* < 0.01 compared with AdBMP2 group. Each assay was done in triplicate and/or carried out in three independent experiments at least; representative results are shown.

To further confirm the role of H19 in BMP2-induced hypertrophic differentiation, we silenced H19 with recombinant adenovirus system and detected the influence of silencing of H19 in BMP2-induced hypertrophic differentiation of MSCs. As for the *in vitro* test, C3H10T1/2 cells infected with AdBMP2, AdBM2 + AdsimH19, and AdGFP were subjected to micromass culture (Figure 4A). Relative RNA expression levels of BMP2 and H19 were tested at day 3, as shown in Figure 4B. AdsimH19 effectively downregulated the expression levels of H19 in AdGFP and AdBMP2 groups without influence expression levels of BMP2 in AdBMP2 and AdBMP2 + AdSimH19 groups. Then, hypertrophic differentiation markers were determined by RT-qPCR and Western blot. As shown in Figures 4C,D, we found that silencing H19 upregulated BMP2-induced Col10α1 and MMP13 expression from day 7 to 12 at genetic level (Figure 4C); meanwhile, the same trend was found at protein level (Figures 4Da,b).

**FIGURE 5:**
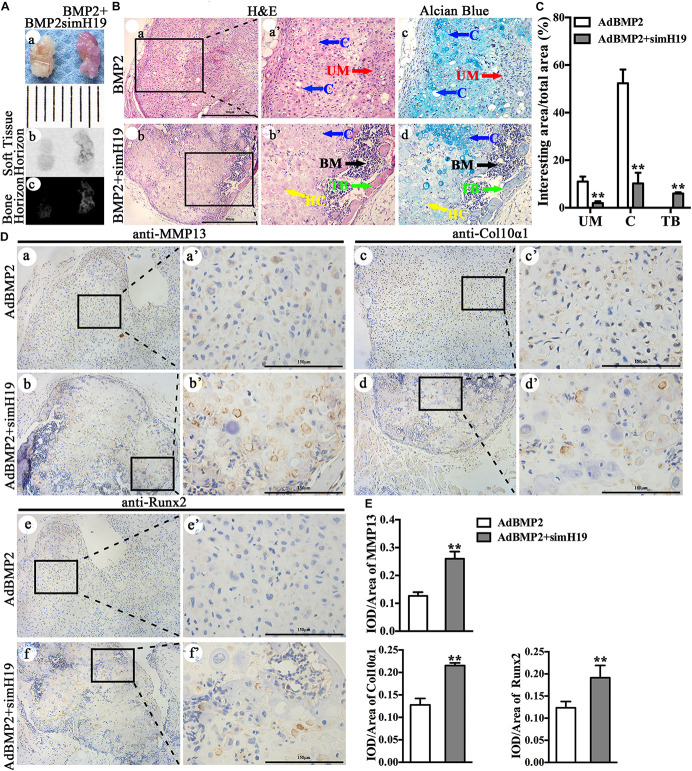
Silencing of H19 promoted bone morphogenetic protein 2 (BMP2)-induced hypertrophic differentiation of mesenchymal stem cells (MSCs) *in vivo*. **(A)** C3H10T1/2 cells infected with AdGFP, AdBMP2, and/or AdsimH19 at the same infection ratio were injected subcutaneously into the flanks of athymic nude (nu/nu) mice for 3 weeks. There was no obvious differences in the morphological phenotype between the cartilaginous/bony masses formed in the AdBMP2 and AdBMP2 + AdSimH19 groups **(a)**, scale = 1 mm; the soft tissue horizon of the masses did not show and obvious difference between AdBMP2 and AdBMP2 + AdSimH19 groups through X-ray testing **(b)**, while the osseous composition in the AdBMP2 + AdSimH19 group was much more than that in the AdBMP2 group through X-ray testing **(c)**. **(B)** On histological examination, masses formed in the AdBMP2 group showed obvious chondrocytes and cartilaginous matrix **(a)**; however, except the chondrocytes and cartilaginous matrix, the masses formed in the AdBMP2 + AdSimH19 group formed obvious trabeculae combined with bone marrow like tissues **(b)**, which indicated the endochondral ossification. At higher magnification, H&E and the Alcian blue staining exhibited less cartilaginous matrix, more hypertrophic chondrocytes, and bone trabecular formation in the AdBMP2 + AdSimH19 group **(b’,c”)** compared with the AdBMP2 group **(a’,a”)**, scale bar = 500 μm. **(C)** Quantitative analysis of undifferentiated MSCs, chondrocytes, and trabecular bone. ImageJ was used to quantitatively analyze undifferentiated MSCs, chondrocytes, and trabecular bone. There were significantly more undifferentiated MSCs, more chondrocytes, and less trabecular bone formation in the AdBMP2 group compared with that in the AdBMP2 + AdSimH19 group. **(D)** The immunohistochemical staining was utilized to confirm the influence of silencing of H19 in BMP2-induced hypertrophic differentiation *in vivo*. The expression of Col10α1, MMP13, and Runx2 in the AdBMP2 group were less and weaken compared with the AdBMP2 + AdSimH19 group **(a,b,c,d,e,f; a’,b’,c’,d’,e’,f’)**. **(E)** Quantitative analysis of positive stained area. Integral optical density/area (IOD/Area) was calculated with Image Pro Plus software. Scale bar = 150 μm. UM, undifferentiated MSCs; C, chondrocytes; TB, trabecular bone. **p < 0.01 compared with AdBMP2 group. Each assay was done in triplicate and/or carried out in three independent experiments at least; representative results are shown.

Using our previously established stem cell implantation assay (Liao et al., 2017a; Zhou et al., 2016), we injected C3H10T1/2 cells infected with AdGFP, AdBMP2, and/or AdsimH19 at the same infection ratio subcutaneously into the flanks of athymic nude (nu/nu) mice for 3 weeks. The cells transduced with AdGFP or AdsimH19 alone failed to form any detectable masses (data not shown). As shown in Figure 5A, there was no obvious morphological differences between the masses formed in the AdBMP2 and AdBMP2 + AdSimH19 group (Figures 5Aa,b). While the osseous composition in the AdBMP2 + AdSimH19 group was much more than that in the AdBMP2 group through X-ray testing (Figure 5Ac). On histological examination (Figure 5B), masses formed in the AdBMP2 group showed obvious chondrocytes and cartilaginous matrix. However, except the chondrocytes and cartilaginous matrix, the masses formed in the AdBMP2 + AdSimH19 group formed obvious trabeculae combined with bone-marrow-like tissues. The Alcian blue staining exhibited that there were less cartilaginous matrix and more hypertrophic chondrocytes formation in the AdBMP2 + AdSimH19 group compared with the AdBMP2 group. In quantitative analysis, we found that in the AdBMP2 group, there were significantly more undifferentiated MSCs (UM) and chondrocytes compared with the AdBMP2 + AdSimH19 group. Moreover, there was significantly more trabecular bone formation in the AdBMP2 + AdSimH19 group compared with AdBMP2 group (Figure 5C).

The immunohistochemical staining was also utilized to confirm the influence of silencing of H19 in BMP2-induced hypertrophic differentiation *in vivo* (Figure 5D). We detected that Col10α1 and MMP13 expression in the AdBMP2 group were less and weakened compared with that in the AdBMP2 + AdSimH19 group (Figures 5Da–d,a’–d’). As the key transcription factor for hypertrophic differentiation, Runx2 expression in AdBMP2 group was also less and weakened compared with the AdBMP2 + AdSimH19 group through immunohistochemical staining (Figures 5De,f,e’,f’). The same trend was found through quantitative analysis (Figure 5E). These data further confirmed that silencing of H19 promoted BMP2-induced hypertrophic differentiation of MSCs *in vivo*.

### H19 Regulate BMP2-Induced Hypertrophic Differentiation of MSCs by Promoting the Phosphorylation of Runx2

**FIGURE 6:**
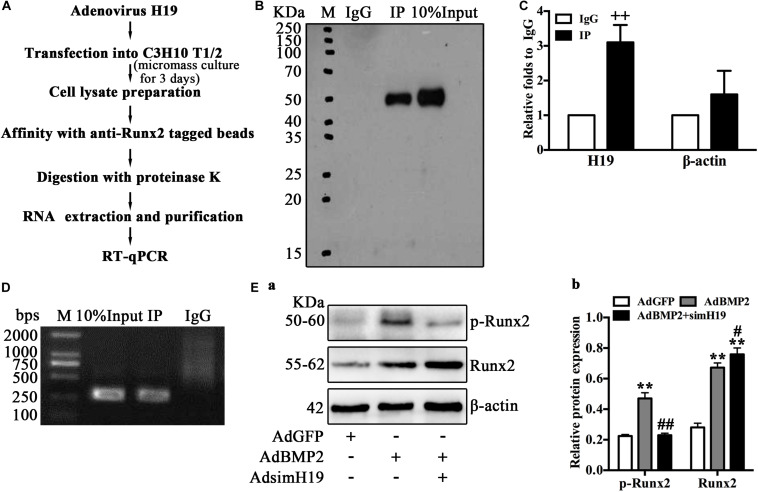
H19 regulates bone morphogenetic protein 2 (BMP2)-induced hypertrophic differentiation of mesenchymal stem cells (MSCs) by promoting the phosphorylation of Runx2. **(A)** Schematic outline of purification of H19-associated ribonucleoprotein (RNPs) and RNA component identification. Briefly, RNA immunoprecipitation (RIP) was performed with mouse monoclonal anti-RUNX2 or preimmune immunoglobulin G (IgG) from extracts of C3H10 T1/2 cells infected with adenovirus expression H19 for 3 days. After immunoprecipitation, proteinase K was used for the enzymolysis of protein and RNA; then, total RNA was extracted, followed by reverse transcription and quantitative real-time PCR (RT-qPCR) and semiquantitative RT-PCR to analyze the combination. **(B)** Immunoprecipitation using anti-RUNX2 or IgG followed by Western blot analysis using a rabbit monoclonal anti-RUNX2. In addition, 10% input was loaded; molecular markers in kDa are on the left. **(C)** H19 levels in immunoprecipitates were determined by RT-qPCR. The levels of H19 and β-actin RNA are presented as fold enrichment in anti-RUNX2 relative to IgG immunoprecipitates. **(D)** Semiquantitative RT-PCR reaction was also used to detect H19 levels in immunoprecipitates, and the PCR products were resolved on 1% agarose gel. **(E)** To assay the posttranscriptional regulation of H19 on Runx2, Western blotting analysis of RUNX2 and phosphorylated RUNX2 expression were performed using a rabbit monoclonal antiphosphorylated RUNX2 (top) and rabbit monoclonal anti-RUNX2 (middle). In bottom, the β-actin was used as control. Molecular markers in kDa are on the left. Western blotting results were quantitatively analyzed by Image Lab software using β-actin as control **(b)** “*” and “**” respectively mean *p* < 0.05 and *p* < 0.01 compared with AdGFP group; “#” and “##” respectively mean *p* < 0.05 and *p* < 0.01 compared with AdBMP2 group; “++” means *p* < 0.01 compared with IgG group. Numbers are mean ± SD (*n* = 3).

As Runx2 is the key transcription factor of BMP2-mediated hypertrophic differentiation of MSCs (Jonason et al., 2009; Liao et al., 2014; Takeda et al., 2001; Ueta et al., 2001; Zhou et al., 2016), we hypothesized that H19 may regulate BMP2-induced hypertrophic differentiation by targeting Runx2. Using RIP analysis, we further analyzed the posttranscriptional regulation of H19 on the phosphorylation of Runx2. The process of RIP analysis is listed in Figure 6A. Briefly, MSCs (C3H10T1/2) infected with adenovirus expression H19 were cultured in micromass; 3 days after infection, cells were lysed and mixed with anti-Runx2 tagged beads. After immunoprecipitation, proteinase K was used for the enzymolysis of protein and RNA; then, total RNA was extracted and purified from the specific RNA protein mixture and subjected to RT-qPCR after to analyze the combination of Runx2 and H19. As shown in Figure 6B, before and after anti-Runx2-tagged beads extraction, Western blot analysis of Runx2 yielded expected products; immunoglobulin G (IgG) was used as control. RT-qPCR analysis showed that, compared with the IgG group, the expression of H19 in the RIP group was significantly higher, and β-actin was used as control (Figure 6C). RT-qPCR products analysis yielded expected products (Figure 6D).

These data strongly suggested the combination of H19 and Runx2; however, how H19 regulates the function of Runx2 is still not clear. As posttranslational modification, especially phosphorylation is one important regulatory mechanism of Runx2 activity, and phosphorylated Runx2 downregulated Runx2 activity and further inhibited Runx2-mediated differentiation (Jonason et al., 2009). Thus, we further ask if H19 influences the phosphorylation of Runx2. As shown in Figure 6Ea, BMP2 upregulated total Runx2 and phosphorylated Runx2. However, BMP2 induced upregulation of total Runx2 and was potentiated by silencing H19, and phosphorylated Runx2 was downregulated dramatically by silencing H19, which indicated that the phosphorylation of Runx2 was blocked with the silencing of H19. Quantitative analysis of the protein band confirmed this trend (Figure 6Eb). This phenomenon indicated that H19 was essential for the phosphorylation of Runx2. Taking these data together, we strongly speculate that H19-mediated phosphorylation of Runx2 regulated BMP2-induced hypertrophic differentiation of MSCs.

## Discussion

Cartilage tissue engineering is potential for the treatment of cartilage pathologies. BMP2 holds the potential to induce MSC chondrogenic differentiation. However, after the chondrocyte formation, BMP2 also stimulates hypertrophic differentiation, which blocks the construction of BMP2-mediated tissue engineering cartilage (Liao et al., 2014; Zhou et al., 2016). Hence, clarifying the mechanisms of BMP2-induced hypertrophic differentiation of MSCs is essential for further application of BMP2-mediated chondrogenic differentiation of MSCs. In the present study, we clarified that physiological expression level of H19 is essential for the phenotype maintaining of BMP2-induced chondrocytes of MSCs. Inhibiting of H19 promotes BMP2-mediated hypertrophic differentiation of MSCs; the mechanisms underlying these processes may be that H19 promotes the phosphorylation of Runx2, which blocks the function of Runx2. These findings applied a version for further construction of BMP2-mediated cartilage tissue engineering.

H19 is a maternal long non-coding RNA in the H19-IGF2 imprint locus, which is abundantly expressed during embryonic development and significantly downregulated after birth (Gabory et al., 2006; Gabory et al., 2009; Gabory et al., 2010; Liu et al., 2017). Although the H19-IGF2 imprinting mechanism has been well clarified (Gabory et al., 2006; Liu et al., 2017), the regulatory functions and mechanisms of H19 in physical and pathological processes are still nebulous. More recently, H19 regulating stem cells differentiation were reported several times. Huang et al. (2016) found that H19 inhibits MSC adipocyte differentiation through epigenetic modulation of histone deacetylases, which indicates that sufficient expression of H19 is necessary to keep MSC osteogenic differentiation. Similarly Liang et al. (2016) characterized that H19 is essential for the osteogenic differentiation of MSCs; overexpression of H19 would accelerate the activation of Wnt/β-catenin pathway and further promote osteoblast differentiation. What is more, Huang et al. (2015) identified H19–miR675–TGF-β1–Smad3–HDAC pathway regulates human bone marrow mesenchymal stem cell (hMSC) osteogenic differentiation, which indicates the prodifferentiation effect of H19. What is interesting is that Dudek et al. (2010) found that the expression of H19 is regulated by key chondrogenic differentiation transcription factor Sox9, and type II collagen expression is regulated by H19-encoded miR675. These researches highly suggested the regulation function of H19 during the process of MSC osteogenic and/or chondrogenic differentiation. What is more, Pang et al. (2019) identified the regulatory function of H19 during MSCs cartilage differentiation. Hence, we focus on the regulatory functions of H19 in BMP2-mediated chondrogenic differentiation of MSCs. We first identified that H19 expression level was relatively high in the proliferative area of mice limb and downregulated in the hypertrophic area of mice limb, which further confirmed the potential role of H19 in promoting cartilage formation. Second, we identified that, with the stimulation of BMP2, peak expression level of H19 was followed after the crest expression of Sox9, which was consistent with the previous study (Dudek et al., 2010). Our further analysis demonstrated that H19 expression level not only positively correlated with the expression level of Sox9 and chondrogenic differentiation markers (Col2α1 and Aggrecan) in the late stage of BMP2 stimulation but also negatively correlated with the expression level of hypertrophic differentiation markers (MMP13, Adamts5, and Runx2). These data indicate that H19 may also function in BMP2-stimulated hypertrophic differentiation, and this hypothesis was identified by our further *in vitro* and *in vivo* tests. To the best of our knowledge, this is the first time to report the regulation function of H19 in hypertrophic differentiation of cartilage. Taken the previous studies and this study together (Dudek et al., 2010; Pang et al., 2019), we deduce that H19 may play a role in cartilage differentiation, cartilage phenotype maintaining, and cartilage hypertrophic differentiation. Hence, appropriate expression level of H19 is essential for the construction of MSC-based cartilage engineering.

Recombinant human bone morphogenetic protein 2 (rhBMP-2) has been approved for treating acute, open tibial shaft fractures and spinal fusion by the Food and Drug Administration (FDA) (Woo, 2013). Our previous work also identified BMP2-induced chondrogenic differentiation of MSCs. However, the mechanisms underlying BMP2-mediated hypertrophic differentiation are far from being clarified. Hypertrophic differentiation following with endochondral ossification is a consecutive process (Hata et al., 2017). In the present study, we first proved that silencing H19 upregulated hypertrophic differentiation markers expression. Second, we confirmed that silencing H19 facilitated BMP2-mediated hypertrophic differentiation and subsequently potentiated BMP2-induced trabecular and bone-marrow-like tissue formation *in vivo*. These data suggested that BMP2-mediated hypertrophic differentiation was regulated by H19. On the other hand, as the key transcription factor of hypertrophic differentiation, Runx2 promotes the maturity of chondrocytes and subsequently regulates hypertrophic differentiation (Jonason et al., 2009; Takeda et al., 2001; Ueta et al., 2001). Hence, clarifying the regulating relation between Runx2 and H19 is extremely urgent for further understanding the mechanisms underlying BMP2-induced hypertrophic differentiation of MSCs. Here, we identified that silencing H19 upregulated the expression of total Runx2 protein level but diminished phosphorylated Runx2 protein level. These results indicated the posttranscriptional regulation function of H19 on Runx2 phosphorylation.

As a novel and effective modulator, H19 was reported to regulate physical and pathological processes in different ways (Zhang et al., 2017). Except as a transregulator of a group of coexpressed genes belonging to the imprinted gene network (Gabory et al., 2010; Lee et al., 2015; Ripoche et al., 1997), H19 also encodes highly reserved microRNA miR675 with exon-1 (Keniry et al., 2012; Raveh et al., 2015; Steck et al., 2012). Meanwhile, lncRNAH19 and miR675 regulate specific biological processes synergistically (Dudek et al., 2010; Muller et al., 2019; Steck et al., 2012). In addition, H19 was identified as a ceRNA that sponged microRNAs and then regulate gene function. For example, H19 antagonizing let-7 microRNA family members (Cao et al., 2019; Kallen et al., 2013), miR-93-5p (Li et al., 2019), miR-17-5p (Liu et al., 2016), miR-107 (Qian et al., 2018), etc. was reported, respectively. Our previous work also found that H19 regulated the expression of microRNAs, which targets Notch signaling pathway (Liao et al., 2017b). What is more, H19 can act as a molecular scaffold to bind with mRNA, then regulate the decay of mRNA (Giovarelli et al., 2014). As for the posttranscriptional regulation, H19 can act as a modular scaffold of histone modification complexes (Tsai et al., 2010). In this study, we inferred the regulation of H19 on the function of Runx2 and first confirmed the combination of H19 and Runx2 through RIP analysis. Then, we identified that silencing H19 could downregulate the phosphorylation of Runx2, which would promote the function of Runx2. Hence, we deduced that H19-mediated phosphorylation of Runx2 regulated BMP2-induced hypertrophic differentiation of MSCs. As an exotic lncRNA, H19 antisense named 91H RNA also reported to play an important role in modulating H19-Igf2 expression, although the exact mechanism remains to be fully understood (Berteaux et al., 2008; Tran et al., 2012). Hence, it is important to further investigate the function of 91H RNA during the process of BMP2-mediated hypertrophic differentiation of MSCs. In addition, on the basis of the current study, it is reasonable to speculate that overexpression of H19 may be beneficial for BMP2-mediated cartilage tissue engineering, which was indicated by Pang et al. (2019). However, as a multifunctional lncRNA, H19 may affect BMP2-mediated chondrogenic differentiation of MSCs and hypertrophic differentiation of cartilage by one or more other mechanisms, such as CeRNA mechanism. Therefore, further *in vivo* cartilage repair test is necessary for identifying the potential of overexpression of H19 for cartilage defect repairing.

In summary, clarifying the mechanisms of hypertrophic differentiation is essential for the construction of BMP2-mediated cartilage tissue engineering. Although several studies have been carried out, the details in regulating BMP2-stimulated hypertrophic differentiation are far from being illuminated. Here, in the aspect of lncRNA, we identified the posttranscriptional regulating function of H19 on Runx2-mediated hypertrophic differentiation, which should be helpful for further construction of BMP2-mediated cartilage engineering.

## Data Availability Statement

All datasets generated for this study are included in the article/Supplementary Material.

## Ethics Statement

The animal study was reviewed and approved by the Ethical Committee of The First Affiliated Hospital of Chongqing Medical University.

## Author Contributions

WH and JL conceived and designed the experiments. GD, JL, HX, and CZ performed the experiments and collected the data. JL, WH, and HC analyzed the data. JL and WH contributed the reagents, materials, and analysis tools. JL, WH, and GD wrote the manuscript. All authors read and approved the manuscript.

## Conflict of Interest

The authors declare that the research was conducted in the absence of any commercial or financial relationships that could be construed as a potential conflict of interest.

## Supplementary Material

FIGURE S1 **(A)** FISH analysis positive control. **(B)** Negative control of IHC.
