# Supplementary figures and images for: LncRNA H19 Regulates BMP2-Induced Hypertrophic Differentiation of Mesenchymal Stem Cells by Promoting Runx2 Phosphorylation
Source: Front Cell Dev Biol. 2020 Jul 29;8:580. doi: 10.3389/fcell.2020.00580 (PMC7438821; doi:10.3389/fcell.2020.00580)

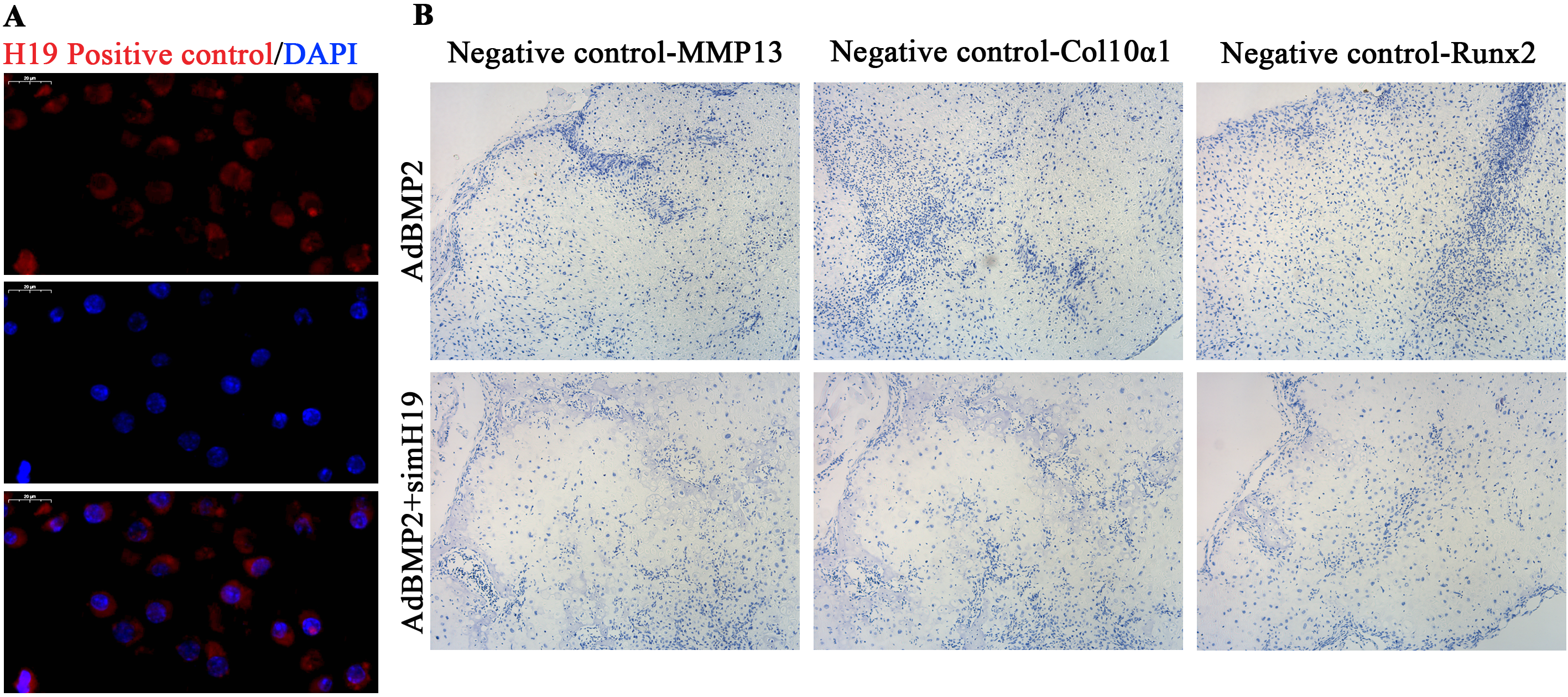

Supplement: FIGURE S1 — (A) FISH analysis positive control. (B) Negative control of IHC. [file Image_1.tif]
